# Barriers and Facilitators to Implementing Interventions for Reducing Avoidable Hospital Readmission: Systematic Review of Qualitative Studies

**DOI:** 10.34172/ijhpm.2023.7089

**Published:** 2023-02-14

**Authors:** Becky Q Fu, Claire CW Zhong, Charlene HL Wong, Fai Fai Ho, Per Nilsen, Chi Tim Hung, Eng Kiong Yeoh, Vincent CH Chung

**Affiliations:** ^1^Centre for Health Systems and Policy Research, Jockey Club School of Public Health and Primary Care, The Chinese University of Hong Kong, Shatin, Hong Kong.; ^2^School of Chinese Medicine, The Chinese University of Hong Kong, Shatin, Hong Kong.; ^3^Department of Medicine, Health and Caring Sciences, Linköping University, Linköping, Sweden.

**Keywords:** Patient Readmission, Transitional Care, Implementation Science, Qualitative Research, Systematic Review, Delivery of Healthcare

## Abstract

**Background:** Avoidable hospital readmission is a major problem among health systems. Although there are effective peri-discharge interventions for reducing avoidable hospital readmission, successful implementation is challenging. This systematic review of qualitative studies aimed to identify barriers and facilitators to implementing peri-discharge interventions from providers’ and service users’ perspectives.

**Methods:** We searched four databases for potentially eligible qualitative studies from databases’ inception to March 2020, and updated literature search for studies published between January 2020 to October 2021. Barriers and facilitators to implementing peri-discharge interventions were identified and mapped onto the Consolidated Framework for Implementation Research (CFIR) constructs. Inductive analysis of the CFIR constructs was performed to yield thematic areas that illustrated the relationship between various facilitators and barriers, generating practical insights to key implementation issues.

**Results:** Thirteen qualitative studies were included in this systematic review. Key issues were clustered in the CFIR constructs of *Design Quality and Complexity* of the intervention, strength of *Network and Communication*, being responsive to *Patient Needs* with sufficient *Resource* support, and *External Incentives*. The three thematic areas were rationality of the interventions, readiness and effort of multidisciplinary implementation teams, and influence of external stakeholders. Common barriers included (*i*) limited resources, (*ii*) poor communication among team members, (*iii*) incompatibility between the new intervention and existing work routine, (*iv*) complicated implementation process, (*v*) low practicality of supporting instruments, and (*vi*) lack of understanding about the content and effectiveness of the new interventions. Common facilitators were (*i*) information sharing via regular meetings on implementation issues, (*ii*) organizational culture that values quality and accountability, (*iii*) financial penalties for hospitals with high avoidable readmissions rates, (*iv*) external support offered via quality improvement programs and community resources, and (*v*) senior leadership support.

**Conclusion:** This study synthesized commonly-presenting barriers and facilitators to implementing peri-discharge interventions among different healthcare organizations. Findings may inform development of implementation strategies in different health systems after appropriate tailoring, based on a consensus-based formative research process.

## Background

 Avoidable hospital readmission is a highly common, costly, and challenging issue in many health systems globally.^1,2^ In the United States, the 30-day all-cause hospital readmission rate was approximately 13.9% in 2016, of which a considerable number is considered as avoidable.^3^ Early hospital readmission is associated with several adverse outcomes, including lower patient satisfaction,^4^ higher risk of mortality,^5^ and evidently increased medical costs and utilization of healthcare services.^5,6^ According to the Global Patient Safety Action Plan 2021-2030, World Health Organization (WHO) recommends investigations on different peri-discharge interventions for reducing the burden of unnecessary hospital readmission.^7^

 A systematic review of 42 trials has shown the beneficial effects of certain peri-discharge interventions for reducing avoidable 30-day hospital readmission. Such interventions are often complex, addressing multiple needs of patients and caregivers,^8^ hence they can be difficult to implement successfully. For instance, a qualitative study among healthcare providers in Denmark indicated that extra multidisciplinary work was required for implementing interventions smoothly on top of routine work, implying additional manpower and cost.^9^ Another qualitative study in the United States suggested that the implementation process is cumbersome as there is a need to integrate services across hospitals, primary and social care.^10^ Multidisciplinary implementation teams led by senior leaders are essential for managing complexities, and for resolving expected conflicts among team members over additional responsibilities.^10^

 Synthesizing different facilitators and barriers of implementing peri-discharge interventions across different health systems would be useful for generating insights on common challenges. Deeper understanding on recurring themes on implementation issues would guide formulation of policy recommendations with higher generalizability. Determinants of implementing peri-discharge interventions for reducing hospital readmission across different healthcare system is yet to be synthesized. We conducted a systematic review to summarize existing qualitative findings concerning barriers and facilitators that influence implementation from the perspectives of different implementers. The implementers included in this systematic review include healthcare providers, social service providers, administrators, or all other personnel who are related to actual implementation. Implications from these findings may inform future development of strategies for implementing peri-discharge interventions effectively.

## Methods

**This** systematic review is reported in accordance with the Enhancing Transparency in Reporting the Synthesis of Qualitative Research (ENTREQ) statement (Supplementary file 1).^11^

###  Eligibility Criteria 

 To be included in this systematic review, a qualitative study should: (*i*) report original results; (*ii*) be published in English; (*iii*) apply qualitative methods for both data collection and data analysis, including but not limited to interviews, focus groups, case studies, ethnographic analysis, and participant observation; (*iv*) include healthcare providers, social service providers, administrators, or other staff who are responsible for implementing peri-discharge interventions; and (*v*) carry an aim of investigating facilitators and barriers of implementing such interventions. We also included mixed-methods studies which used both qualitative and quantitative methods, given that data originating from qualitative methods are adequately reported for extraction and synthesis. We excluded review articles, protocols, conference abstracts, scientific statements, or workshop reports. Studies which did not report qualitative results were also excluded.

###  Literature Search

 We searched for qualitative studies in four international electronic databases (EMBASE, MEDLINE, PsycInfo and Global Health) from their inception to March 2020 (Supplementary file 2). As research on readmission reduction interventions evolve rapidly, we updated the search for potentially eligible studies in these four databases in the period from March 2020 to October 2021. This allowed us to include newly eligible studies, ensuring that the results are thorough and up to date (Supplementary file 3). The search strategy was tailored to each database using a combination of MeSH terms and keywords to cover the concepts of “peri-discharge interventions” and “hospital readmission.” Details could be found in Supplementary files 2 and 3. Specialized filters with maximized sensitivity for qualitative study were applied in MEDLINE, EMBASE, and PsycInfo.^12,13^ No restrictions on publication status were imposed.

###  Literature Selection 

 Two reviewers (BQF and CCZ) independently screened titles and abstracts of potential studies and assessed full text for eligibility. Disagreements were resolved by discussion and consensus between the two reviewers. A third reviewer (VCC) was consulted to settle unsolved disagreement.

###  Methodological Quality Assessment 

 Methodological quality of all included qualitative studies was assessed using the Critical Appraisal and Skills Programme (CASP) checklist.^14^ It includes 10 specific questions on methodology including aims of the research, qualitative methodology, research design, recruitment strategy, data collection approach, data analysis, researcher-participant relationship, ethical issues, statement of findings and research value. Each question was answered, based on information reported in the publications, using one of the following responses: ‘yes,’ ‘no,’ or ‘can’t tell.’ CASP does not provide a quantitative scoring scheme for appraising methodological quality.^14^ Methodological limitations of each aspect for each study were identified accordingly. Methodological quality assessment was conducted independently by two reviewers (BQF and CCZ). Discrepancies were resolved through discussion and consensus-building between the two reviewers. A third reviewer (CHW) was consulted for unresolved disagreement.

###  Data Extraction and Analysis

 Two reviewers (BQF and CCZ) used a pre-designed data extraction form to collect the following information from each included study independently: first author, year of publication, study location, study aim, nature of peri-discharge interventions, data collection method, data analysis method, type of participants, sample size, and qualitative results for further analysis.

 In this systematic review, extracted qualitative results were analyzed using a framework synthesis approach.^15,16^ This approach begins with the use of a pre-existing framework for initial deductive coding of data, which is then followed by inductive analysis focusing on identifying emerging new themes. Based on overall synthesis findings, key thematic areas relevant to implementation were then identified.^17^

**Figure 1:**
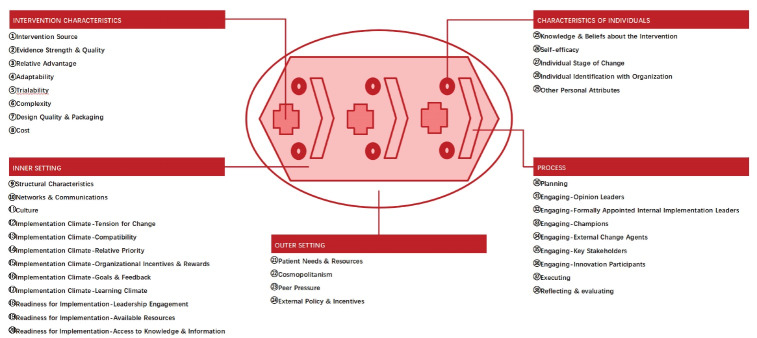
Schematic Diagram of the Consolidated Framework for Implementation Research.

 Consolidated Framework for Implementation Research (CFIR) is selected as the initial coding framework, as it can be used as a standardized structure for synthesizing qualitative findings associated with implementation barriers and facilitators in a comprehensive manner.^18,19^ The CFIR comprises a set of constructs which can be applied in diverse scenarios and settings, including healthcare providers’ experiences in implementing interventions.^20^ There are 38 constructs across five domains in the CFIR, namely intervention characteristics, inner setting, outer setting, characteristics of individuals, and implementation process.^21^ Each construct can function as a barrier and/or facilitator to implementation, either influencing implementation negatively, ie, making implementation more difficult (ie, acting as a barrier), or influencing implementation positively, ie, making implementation easier (ie, acting as a facilitator).^18-20^ The domains and constructs should not be considered in isolation of each other, as complex interactions among domains and constructs may influence the implementation of interventions.^18,20^ A schematic diagram of the CFIR is shown in Figure 1.

 Firstly, deductive coding based on the CFIR was performed. Two reviewers (BQF and CCZ) independently coded findings reported in each included study into various CFIR constructs using NVivo software after co-piloting.^22^ A third reviewer (VCC) was consulted to settle unresolved disagreements between coders. To facilitate the identification of commonly reported barriers and facilitators, the number of included studies which described specific CFIR constructs was analyzed. Constructs that were described in three or more included studies were considered as commonly reported CFIR constructs associated with implementation of peri-discharge interventions.

 Secondly, inductive analysis of the CFIR constructs was performed to yield thematic areas, of which such synthesis aimed to illustrate relationships between various facilitators and barriers to implementation. In this part, the authors (VCC, BQF, and CCZ) conducted an interpretation on the relationships between themes from the first part of the analysis. Interpretation on the linkage between the CFIR constructs of external policy and incentive, intervention participants and cosmopolitanism in the context of peri-discharge intervention were performed. Their relationships suggested that external stakeholders, including patients, caregivers as well as policy makers would have strong influence on implementation outcomes, and their involvement in the implementation process may improve chances of success. Findings from inductive analysis were critically reviewed by all authors prior to finalization. Findings were then synthesized into various CFIR constructs under each thematic area.

## Results

###  Study Selection

**Figure 2:**
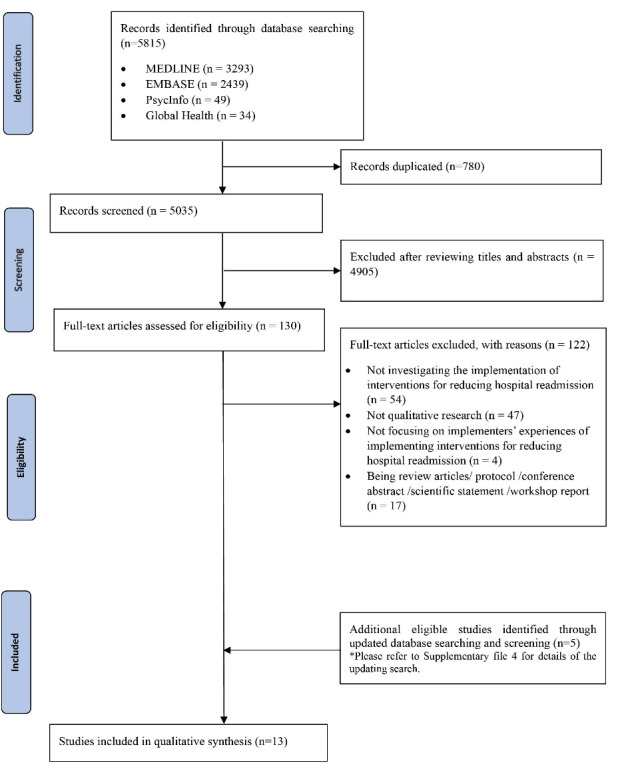
Flowchart of Literature Search and Selection.

 Among the 5815 records obtained through two literature searches, 780 duplicates were identified and excluded. After screening titles and abstracts, 4905 citations were excluded. Full-text articles of the remaining 130 citations were retrieved for further assessment, of which 122 publications were excluded due to the following reasons: not investigating the implementation of interventions for reducing hospital readmission (n = 54); not being qualitative study (n = 47); not focusing on implementers’ experiences (n = 4); and being review articles, protocols, conference abstracts, scientific statements, and workshop reports (n = 17). Updated literature search for potential qualitative studies from March 2020 to October 2021 identified 5 additional studies that were considered eligible (Supplementary file 4). A total of thirteen qualitative studies,^9,10,23-33^ were included. Details of literature search and study selection are presented in Figure 2.

###  Study Characteristics

 All included studies were published between 2013 and 2021. Ten studies were conducted in the United States, and the other three were conducted in Denmark, Norway, and Singapore. Five included studies derived data from individual interviews, while two obtained data from document analysis and individual interviews. Three studies collected data via focus group interviews, and three used both individual and focus group interviews. Eight studies utilized thematic analysis, while two utilized framework analysis. The remaining studies were based on grounded theory (n = 1) and content analysis (n = 2). One included study investigated case managers’ experiences only, and one focused on nurses only. The remaining eleven included studies explored views from at least three different types of implementers. Amongst these eleven studies, nurses were the most frequently investigated professional category (n = 8), followed by physicians (n = 6) and administrators (n = 5). Detailed characteristics of included studies are presented in Table 1.

**Table 1:** Characteristics of 13 Included Qualitative Studies

**First Author, Year of Publication, Region**	**Study Aim**	**Peri-discharge Interventions**	**Data Collection Method**	**Methods of Qualitative Analysis**	**Role of Participants** **(No. of Participants)**
Danielsen 2020, Norway^23^	To understand why readmission reduction intervention failed in some aspects while succeeded in others from a nursing perspective. Topics included: (1) appropriateness of the intervention dose (ie, number of days and calls administered) and fidelity of the intervention; (2) mechanisms of positive/negative impacts; and (3) contextual factors that may have influenced the intervention in unanticipated ways.	Telephone intervention, consisting of 30 days of continuous phone-support (hotline) and two scheduled phone-calls after discharge following surgical aortic valve replacement.	Focus group interviews	Content analysis	Nurses. Detailed number of participants were not reported.
Lai 2021, Singapore^24^	To examine the challenges and lessons in implementing a holistic care model at a regional acute hospital and its community partners for reducing readmission.	A specialist-led general medicine care model, implementing proper discharge planning at an acute hospital, conducting post-discharge home visits, and providing medical support to institutionalized patients in the community.	Individual interviews and focus group interviews	Thematic analysis	Individual interview: Clinicians (n = 8), administrators (n = 10). Focus group interview: Clinicians (n = 3), administrators (n = 3).
Lee 2013, US^25^	To understand the perspectives of physicians, nurses and social workers in the process of implementing interventions for reducing readmission in a large academic medical center.	Care transition program, emphasizing accountability, communication, and involvement of the patient and family members in plans of care.	Individual interviews and focus group interviews	Grounded theory	Individual interview: physicians (n = 24), nurses (n = 5). Focus group interview: physicians (n = 9), nurses (n = 13), social workers (n = 6).
Lehn 2018, Denmark^9^	To examine the experiences of physicians, nurses, medical secretaries and administrators that work with implementing readmission prevention program for elderly patients in five different hospitals.	A post-discharge follow-up program, in which nurses and GPs conduct joint visits in patients’ homes, reviewing their treatment plans, functional levels, environment, and current medicine intake, and then planning ongoing care.	Focus group interviews	Framework analysis	Physicians (n = 6), nurses (n = 11), medical secretaries (n = 3), administrators (n = 4).
Machta 2016, US^26^	To identify barriers and facilitators to implementing interventions for reducing hospital readmission from the perspectives of case managers, pharmacists, physicians, nurses and other supporting staff in a large academic medical center.	Care transition program, containing a multicomponent and multidisciplinary pre-discharge services including: (1) needs assessment by case managers, (2) medication history by medication transition specialists (pharmacy technicians), (3) medication reconciliation and counseling by pharmacists, (4) communication to outpatient provider by physicians, and (5) self-care education using teach-back and scheduling of timely follow up by nurses.	Individual interviews	Framework analysis	Case managers (n = 3), pharmacists (n = 6), physicians (n = 6), nurses (n = 8), other supporting staff (n = 2).
Meehan 2017, US^27^	To explore challenges to implementing interventions for reducing hospital readmission among healthcare providers, social service providers and community leaders in fifteen communities.	Statewide collaboration interventions, including statewide education on quality improvement strategies and community-specific technical assistance on collaboration approaches in the delivery of peri-discharge interventions.	Documents analysis and individual interviews	Thematic analysis	Healthcare providers, social service providers and community leaders. Detailed number of participants were not reported.
Meehan 2015, US^28^	To identify barriers and suggestions for implementing interventions for reducing hospital readmission among administrators, nurses and other supporting staff in five skilled nursing facilities.	A quality improvement project for the delivery of peri-discharge interventions. Providing training and technical assistance to administrative and clinical staff of skilled nursing facilities.	Documents analysis and individual interviews	Thematic analysis	Administrators, nurses, other supporting staff. Detailed number of participants were not reported.
Misra-Hebert 2021, US^29^	To examine providers’ experiences in participating in a post-discharge home visit program for patients at high risk for readmission.	A post-discharge home visit program, providing home visits with standardized medical record and extra telephone follow ups.	Individual interviews	Thematic analysis	Registered nurses (n = 7), primary care physicians (n = 9), paramedics (n = 3), advanced practice registered nurses (n = 3).
Mitchell 2016, US^10^	To understand the experience of implementing interventions for reducing hospital readmission from the perspectives of organizational leaders, administrators, physicians, nurses, case managers, pharmacists and other supporting staff in ten different hospitals.	A Re-Engineered Discharge (Project RED) program, delivering a patient-tailored hospital discharge plan to improve safety during care transition.	Individual interviews	Thematic analysis	Organizational leaders, administrators, physicians, nurses, case managers, pharmacists, other supporting staff. Detailed number of participants were not reported.
Nation 2019, US^30^	To explore case managers’ perceptions of implementing interventions for reducing hospital readmission among elderly in a managed care organization.	Discharge planning, a multidisciplinary approach to prepare and assist patients and their families as they move to the next level of care outside of hospital.	Individual interviews	Content analysis	Case managers (n = 9).
Rask 2017, US^31^	To identify contextual factors which influence the implementation of interventions for reducing readmission among organizational leaders, administrators, nurses and other supporting staff in three skilled nursing facilities.	Interventions to Reduce Acute Care Transfers II, a set of evidence-based clinical practice tools and strategies directed toward residents of long-term care settings, including quality improvement tools, communication tools, decision support tools, and advanced care planning tools.	Individual interviews	Thematic analysis	Organizational leaders, administrators, nurses, other supporting staff. Detailed number of participants were not reported.
Riddle 2020, US^32^	To elicit suggestions for improving a nurse visit intervention for reducing hospital readmission.	A post-discharge home visit, containing a single home visit from a registered nurse within 96 hours of discharge.	Focus group interviews	Thematic analysis	Primary care physicians (n = 7), hospital medicine physicians (n = 12), registered nurses (n = 10).
Romaire 2020, US^33^	To explore successes, challenges, and lessons learned in implementing a readmission reduction program. Topics included practice transformation, use of health IT and data analytics, and integration with primary care.	Medicaid behavioral health homes, providing multidisciplinary team-based care, enhanced access to care, population risk stratification and management, patient- and family-directed care plans, promoting decision support, optimizing the capacity of clinical information systems, as well as integrating general medical and behavioral healthcare by partnering with patients primary care providers.	Individual interviews and focus group interviews	Thematic analysis	Individual interviews: the funders, leadership, state officials, commercial payers, and healthcare service providers. Detailed number of participants were not reported. Focus group interviews: healthcare service providers. Detailed number of participants were not reported.

Abbreviation: GPs: general practitioners.

###  Methodological Quality of Included Studies 

 Results of methodological quality assessment are presented in Supplementary file 5. All included studies stated the aims of research clearly, used appropriate qualitative methodology, collected data in a manner that addressed the research issue, provided clear statements of findings, and demonstrated the research value. Among the thirteen included studies, only one failed to conduct a sufficiently rigorous data analysis. Six did not use appropriate research designs and four did not clearly state the recruitment strategies. Five studies did not make clear statements on potential ethical issues, and nine did not adequately consider bias which may arise from relationships between researchers and participants.

###  Barriers and Facilitators

 The reporting frequency of barriers and facilitators aligned to the CFIR constructs, and synthesized findings summarized under CFIR are reported in Tables 2 and 3, respectively. Frequently highlighted implementation issues across included studies were concentrated in the CFIR constructs of *Design Quality and Packaging, Complexity* of the intervention, strength of *Network and Communication*, being responsive to *Patient Needs* with sufficient *Resource* support, and *External Incentives* including both support and penalties.

**Table 2:** Frequency Table of Cited Consolidated Framework for Implementation Research Constructs (n= 13 Studies)

**CFIR Domains and Constructs**	**Barriers, No. of studies (%) **	**Facilitators, No. of studies (%)**
Intervention characteristics		
Intervention Source	0 (0)	0 (0)
Evidence strength and quality	0 (0)	2 (15)^26,30^
Relative advantage	0 (0)	1 (8)^26^
Adaptability	2 (15)^29,32^	2 (15)^26,27^
Trialability	0 (0)	0 (0)
Complexity	4 (31)^9,10,25,33^	0 (0)
Design quality and packaging	4 (31)^9,10,24,26^	2 (15)^23,32^
Cost	1 (8)^33^	0 (0)
Outer setting		
Patient Needs & Resources	5 (39)^24,25,29,30,32^	3 (23)^29,30,32^
Cosmopolitanism	3 (23)^10,24,32^	0 (0)
Peer pressure	0 (0)	0 (0)
External policy and incentives	0 (0)	4 (31)^10,26,31,33^
Inner setting		
Structural characteristics	2 (15)^9,25^	0 (0)
Networks and communications	5 (39)^9,10,25,26,33^	8 (62)^10,23,25-27,30,32,33^
Culture	1 (8)^10^	3 (23)^10,26,28^
Implementation climate		
Tension for change	0 (0)	0 (0)
Compatibility	3 (23)^9,26,31^	0 (0)
Relative priority	0 (0)	1 (8)^26^
Organizational incentives and rewards	0 (0)	0 (0)
Goals and feedback	0 (0)	1 (8)^26^
Learning climate	1 (8)^26^	0 (0)
Readiness for implementation		
Leadership engagement	1 (8)^27^	3 (23)^10,27,28^
Available resources	5 (39)^9,24-26,28^	0 (0)
Access to knowledge & information	1 (8)^26^	4 (31)^23,26,30,33^
Characteristics of individuals		
Knowledge & beliefs about the intervention	3 (23)^9,10,26^	2 (15)^26,32^
Self-efficacy	1 (8)^24^	0 (0)
Individual stage to change	0 (0)	0 (0)
Individual identification with organization	2 (15)^10,31^	0 (0)
Other personal attributes	0 (0)	0 (0)
Process		
Planning	2 (15)^9,10^	0 (0)
Engaging		
Opinion leaders	0 (0)	0 (0)
Formally appointed internal implementation leaders	0 (0)	0 (0)
Champions	0 (0)	0 (0)
External change agent	0 (0)	2 (15)^31,33^
Key stakeholder	3 (23)^24,32,33^	1 (8)^32^
Intervention participant	5 (39)^24,25,29,30,32^	2 (15)^29,30^
Executing	0 (0)	0 (0)
Reflecting and evaluating	2 (15)^24,26^	2 (15)^10,27^

Abbreviation: CFIR, Consolidated Framework for Implementation Research.

**Table 3:** Summary of Findings Summarized Under CFIR Constructs

	**Barriers of Implementation**	**Facilitators of Implementation**
Intervention Characteristics		
Evidence strength and quality	N/A	Evidence-based guideline facilitated the implementation of interventions for reducing hospital readmission with a standardized process.^30^
	N/A	High quality evidence supporting the effectiveness of interventions for reducing hospital readmission would facilitate healthcare providers’ implementation.^26^
Relative advantage	N/A	If implementing interventions for reducing hospital readmission could bring additional benefits on top of routine practice, healthcare providers would be more willing to change their current practice.^26^
Adaptability	Standardized interventions may be suitable for most patients in routine care, but these may not match specific needs among patients with certain diseases.^29,32^	Implementation process would be improved by tailoring the delivery of interventions for reducing hospital readmission according to local contexts and needs.^26,27^
Complexity	Interventions for reducing hospital readmission usually contained multiple components. The increased complexity of interventions would make the implementation process cumbersome.^9,10,25,33^	N/A
	Delivery of interventions for reducing hospital readmission would impede the routine discharge workflow.^10^	N/A
Design quality and packaging	Operational defects of supporting instruments (eg, IT systems, implementation checklists, etc)^10^ Delayed interdisciplinary coordination process due to the use of suboptimal digital communication systems.^9^	Appropriate supporting guidelines and clinical pathway can improve workflow efficiency and effective communication among implementers.^23,32^
	Nurses and managers indicated that suboptimal implementation checklist items may not map to a specific clinical workflow.^26^	N/A
	If tools for evaluating patients’ condition is not standardized, workflow and decision making is wholly dependent on healthcare professionals’ judgement. Variations in individual judgement affected outcome of the readmission reduction interventions.^24^	N/A
Cost	Operating and maintenance costs of the supporting system pose a challenge to intervention implementation.^33^	N/A
Outer setting		
Patient needs and resources	Inadequate family, financial and social support would pose negative influence on patients' post-discharge care.^24,25,29,30,32^	Understanding patient's personal and family circumstances, as well as their needs, would facilitate the planning and implementation of interventions for reducing hospital readmission.^29,30^
	Inaccuracy of patients' self-reported information would increase nurses' difficulties in identifying patient needs.^25,30^	Good family support would help patients to better comply with the intervention.^29^
	Patients with mental health issues, as well as those who have high expectations on healthcare professionals and hospital care, may not accept the intervention.^24,32^	Implementing the interventions with input from patients and caregivers allowed them to have a sense of ownership and collaboration. As stakeholders, their confidence about the intervention would hence increase.^32^
Cosmopolitanism	One hospital decided against implementing follow-up appointments to hospital-owned services since it might be viewed as competitive by physicians in community practice.^10^	N/A
	Loose connections between the hospital, primary care and social care organizations would result in barriers to information sharing, and subsequently affecting care coordination.^24,32^	N/A
External policy and incentives	N/A	Promulgation of financial penalties for hospitals with high readmission rates could urge healthcare providers to implement interventions for reducing readmission.^10^
	N/A	External support offered via quality improvement programs can encourage healthcare and social service providers to implement interventions for reducing hospital readmission.^26^
	N/A	Sufficient funding and a reimbursement model accepted among intervention providers would promoted smooth implementation.^31,33^
Inner setting		
Structural characteristics	Workflow and structure of various departments involved in implementing the interventions were not coordinated. The implementation process was thus incoherent.^9^	N/A
	Implementation often requires inter-departmental support. Complexity and fragmentation of health and social care delivery systems would exacerbate difficulties in inter-departmental cooperation.^25^	N/A
Networks and communications	Inadequate and ineffective communication in the multidisciplinary implementation teams hindered the information exchange.^10,25,33^	Regular meetings and discussion in the multidisciplinary implementation teams allowed them to share latest information related to patients' discharge process with each other.^10,27^
	Misunderstandings among multidisciplinary team members would lead to poor cooperation on the implementation process.^9,26^	Multidisciplinary collaboration would strengthen the quality stability, adaptability and sustainability of intervention implementation.^25,26^
	N/A	Close patient follow-up and timely information sharing between different providers are considered useful.^23,30,32,33^
Culture	Implementation of interventions for reducing hospital readmission often includes changes in routine practice. Conservative organizational culture may contribute to a dominance of resistance to change among healthcare providers.^10^	An organizational culture that valued quality improvement and accountability was essential for successful implementation.^10,26,28^
Implementation climate		
Compatibility	Excessive workload brought by implementation of additional interventions for reducing hospital readmission would make providers feel exhausted.^26,31^	N/A
	Additional tasks would be required for implementing interventions for reducing hospital readmission. This might create tensions with existing workload among healthcare and social service providers.^9,26^	N/A
Relative priority	N/A	When interventions for reducing readmission was recognized as a priority by the hospitals, healthcare providers would be more willing to implement the interventions.^26^
Goals and feedback	N/A	Regular feedback enhanced healthcare and social service providers’ motivation of implementing interventions and helped them set goals.^26^
Learning climate	Overlooking nurses’ efforts in learning how to improve the intervention would lower their motivations of implementation.^26^	N/A
Readiness for implementation		
Leadership engagement	Lack of support from senior leaders in the organizations would restrict implementation.^27^	Senior leaders would support their colleagues in integrating the interventions into routine practice.^10,27,28^
Available resources	Limited resources (eg, a lack of manpower, diagnostic resources, training for multidisciplinary implementation team and time for interdisciplinary communication) might hinder different steps of implementation process.^9,24-26,28^	N/A
Access to knowledge and information	Lack of formal training on how to implement the intervention may lead to confusion among nurses in the process.^26^	Provision of tailored post-discharge services requires detailed needs assessment during the discharge planning process.^30^
	N/A	Training providers on standardized intervention procedures increases confidence, and this can enable them to make timely adjustments for ensuring intervention fidelity.^23,26,33^
Characteristics of individuals		
Knowledge and beliefs about the intervention	Physicians, nurses and case managers' lack of understanding about the interventions, and doubts concerning the interventions effectiveness would reduce the enthusiasm for implementation.^9,10,26^	A better understanding on the differences between interventions for reducing hospital readmission and usual care among healthcare and social service providers would facilitate the implementation.^32^
	Some physicians viewed the standardized approach of implementing the interventions as a threat to professional autonomy.^9,26^	Familiarity with details of the intervention may improve acceptability among providers, promoting active implementation.^26^
Self-efficacy	General practitioners may have no confidence in their capacity in handling complex patient cases discharged from hospitals.^24^	N/A
Individual identification with organization	Multidisciplinary implementation team members may perceive that the hospital management underestimated the difficulty of delivering the interventions.^10^	N/A
	Physicians' low commitment to the organization discouraged other healthcare and social service providers to implement the interventions.^10,31^	N/A
Process		
Planning	Plans that underestimate healthcare providers’ workload would cause practical problems during implementation.^10^	N/A
	Plans without clear division of labour would hinder cooperation among healthcare and social service providers.^9^	N/A
Engaging		
External change agents	N/A	Technical assistance from experienced external advisors, consultants and quality improvement organizations can often provide professional support to new providers.^31,33^
Key stakeholders	Lack of consensus among key stakeholders could led to unclear roles and responsibilities of among implementers themselves, or among external partners. This may have a negative impact on the team’s workflow.^24,32,33^	N/A
Intervention participant	Poor communication between providers, patients and their caregivers would pose negative influence on patients' confidence and compliance towards the interventions.^30,32^	N/A
	Some patients failed to understand the discharge instructions from providers comprehensively.^24,25,29,30^	Promoting patients’ active participation can reduce unnecessary trivial work on the providers’ side. Nurses’ role in educating patients and caregivers on implementation details, and serving as coordinators can help service recipients to better understand and accept interventions.^29,30^
Reflecting and evaluating	Delayed feedback might make providers feeling frustrated as their performance with current mode of delivery remain unclear.^26^	Formative and summative assessments on the implementation process would facilitate targeted improvement in delivery process.^10,27^
	Concerns with inaccuracy of performance-based evaluation of the intervention may worry the providers as they this may undermine their credit in delivering the intervention.^24^	N/A

Abbreviations: CFIR, Consolidated Framework for Implementation Research; N/A, not applicable.

 After identified all possible barriers and facilitators from included studies (Table 3), three thematic areas spanning across these CFIR constructs were established to facilitate interpretation, including (*i*) rationality of the interventions, (*ii*) readiness and effort of multidisciplinary implementation teams, and (*iii*) external stakeholders (Table 4). For example, the construct of evidence strength and quality is a key element in the theme of rationality of the interventions, as clinical guidelines supported by high-quality evidence were essential for justifying healthcare professionals’ behavior in implementing new interventions. Amongst the three thematic areas, sixteen commonly reported CFIR constructs, which were described in at least three included studies, were further elaborated in the following sections.

**Table 4:** Overarching Thematic Areas Spanning Across Consolidated Framework for Implementation Research Constructs

**Thematic Areas**	**Description **	**CFIR Construct (CFIR Domain)**
Rationality of the interventions	This theme refers to the rationale and operability of intervention designs, focusing on the characteristics, support conditions as well as components of the interventions themselves. This included compatibility with the context and the practicality of supporting instruments and implementation processes.	Evidence strength and quality *(intervention characteristics)* Relative advantage *(intervention characteristics)* **Complexity** *(intervention characteristics)* **Design quality and packaging** *(intervention characteristics)* **Compatibility** *(inner setting)* Goals and feedback *(inner setting)* Planning* (process)*
Readiness and effort of the multidisciplinary implementation teams	Organizations, healthcare and social service providers who implemented interventions were committed to change routine practice during the preparation and implementation phases. This included the establishment of internal mechanisms for support, personal awareness about the interventions and availability of execution plans.	**Adaptability** *(intervention characteristics)* Cost* (intervention characteristics)* Structural characteristics *(inner setting)* **Networks and communications** *(inner setting)* **Culture** *(inner setting)* Relative priority *(inner setting)* Learning climate *(inner setting)* **Leadership engagement** *(inner setting)* **Available resources** *(inner setting)* **Access to knowledge and information** *(inner setting)* **Knowledge and beliefs about the intervention** *(characteristics of individuals)* Self-efficacy *(characteristics of individuals)* Individual Identification with organization *(characteristics of individuals)* **Key stakeholders** *(process-engaging)* Intervention participant – provider-patient communication *(process)* **Reflecting and evaluating** *(process)*
External stakeholders	Involvement of patients and their caregivers, as well as all external providers and parties that influenced the process of implementation.	**Patient needs and resources** *(outer setting)* **Cosmopolitanism** *(outer setting)* **External policy and incentives** *(outer setting)* External change agents (process-engaging) **Intervention participant** – patients confidence and compliance *(process)*

Abbreviation: CFIR, Consolidated Framework for Implementation Research. Note: Constructs in bold were commonly reported constructs, which were described in at least three included studies. These commonly reported constructs were further introduced in the following sections.

####  Rationality of the Interventions

 This theme refers to the rationale and operability of peri-discharge intervention designs, focusing on the characteristics, components and necessary support needed for implementing this complex intervention successfully.

**Complexity:** Interventions for reducing hospital readmission are usually comprised of multiple components across hospital, primary care and social services.^10,33^ These additional interventions increase the workload, and disrupt the routine discharge process of the hospitals.^9,10^ The complex nature of peri-discharge interventions is a barrier as the implementation process could be cumbersome and complicated.^9,10,25,33^

**Design quality and packaging:** Different supporting instruments, such as software plug-in to electronic health records, implementation checklists and service delivery guidelines, were developed to facilitate the delivery of interventions in different organizations.^23,32^ However, these innovations may not fit in existing infrastructure. Some hospitals reported difficulties in integrating peri – discharge intervention application software with existing electronic health records.^10^ Another example is that the use of new software may cause delayed communications due to unfamiliarity, hampering the multidisciplinary coordination process.^9^ Besides, nurses and pharmacists indicated that additional tasks demanded in the peri-discharge intervention implementation checklist may not be compatible with routine workflow.^24,26^ In general, innovations which are meant to assist implementation may indeed be recognized as barriers by implementers due to limited usefulness, or lack of integration with existing clinical pathways.

**Compatibility:** Since additional tasks would be required for implementing peri-discharge interventions, the workload of healthcare professionals would be increased.^9,31^ Indeed, heavy workload was a common complaint made by healthcare professionals, especially when additional work were not compatible with established discharge routines.^9,31^ Such incompatibility would cause tensions or even conflict among implementation leaders and frontline healthcare professionals, posing a significant hurdle to the change process.^9,26^

####  Readiness and Effort of Multidisciplinary Implementation Teams

 This theme relates to how organizations, healthcare and social service providers are prepared for, and actually changing the routine practice to enable implementation of peri-discharge interventions.

**Adaptability: **Due to the diverse local contexts and different needs of individual patients and caregivers, healthcare professionals often consider peri-discharge interventions should not be standardized.^29,32^ The intervention design and implementation process needs to be adapted to fit organizational and individual needs. The scope and complexity of tailoring such complex intervention may vary according to the local contexts in different healthcare systems.^26,27^ This adaptation process represents another barrier to implementation for healthcare and social services providers, as extra tasks of need assessments and intervention modifications would add to the workload.

**Networks and communications:** The multidisciplinary implementation team usually comprises healthcare and social service providers working in different organizations. Inadequate and ineffective communication among the team members can hinder information exchange.^9,25,33^ Inaccurate communication would subsequently lead to poor cooperation in the implementation process.^9,25,30,33^ To overcome this barrier, leaders and managers found that regular meetings and discussions, as well as usage of shared electronic health record system can promote timely communication across all parties.^9,10,27,30,33^ Efficient multidisciplinary communication is believed to enhance the stability and adaptability of the process, improve the quality and sustainability of implementation, and therefore function as a facilitator.^23,25,32^

**Culture and leadership engagement:** Organizational culture which value quality improvement and accountability is considered as a key for successful implementation.^10,26,28^ Meanwhile, a conservative organizational culture might contribute to healthcare providers’ resistance to change.^10^ This require proper handling from the senior management as the implementation of a new intervention almost always mandates changing the routine practice.^10^ Given the cross – organizational nature of peri-discharge intervention, leadership support from different organizations involved in service provision is needed to facilitate effective implementation. Involving senior leaders in the implementation team could promote the use of novel interventions by subordinate team members internally, and also directly facilitate collaboration externally with other organizations.^34^

**Available resources:** Physicians, social workers, case managers and administrators reported a lack of resources to support additional work was a major implementation barrier.^9,24-26,28^ For instance, insufficient manpower and training for a multidisciplinary implementation team, as well as limited time for communicating among team members, can hinder multiple steps in the implementation process.^9,25,26,28^

**Access to knowledge and information:** Nurses are often considered as the key link in the implementation process as they act as the “super connector” between patients, caregivers, different providers and organizations, coordinating flows of information required for successful implementation. Their practical knowledge on peri-discharge interventions’ details is essential for their coordinating role, as other team members often depend on nurses for knowledge and information to decide how the intervention should be delivered based on patients’ need.^26,33^

**Knowledge and beliefs about the intervention:** Knowledge on the effectiveness and value of the intervention is important for acceptance among physicians and nurses, motivating commitment to change current routine and implementing a new peri-discharge intervention.^9,10,26,32^ Echoing the need for adaptation, standardized peri-discharge intervention strategies may be perceived as inappropriate among physicians. This belief may hinder implementation unless flexibility in intervention component is allowed,^9^ but the tailoring process per se can subsequently increase implementation burden.

**Key stakeholders:** In the multi-disciplinary delivery of peri-discharge interventions, all providers are indeed key stakeholders. When organizations and implementers fail to reach a consensus on each other’s roles and responsibilities, it is difficult for the whole team to cooperate.^24,32,33^ Nurses could have a key role in fostering consensus across different organizations and providers, integrating different components of the peri-discharge interventions into a coherent workflow across hospital, primary care, social services, patients and caregivers.^32^

**Reflecting and evaluating:** Delayed feedback on how the intervention reduces readmission might make some case managers feel frustrated, as they became uncertain about the real-world effectiveness of peri-discharge intervention.^26^ Meanwhile, clinicians may worry about the inaccuracy of performance indicators, as such inaccuracy may discredit their efforts in implementing the new intervention.^24,26^ If the performance indicators are credible and acceptable among frontline providers, organizational leaders and administrators generally agreed that formative and summative assessments would facilitate improvement in implementation quality.^10,27^

####  External Stakeholders

 This theme refers to the involvement of patients and their families, as well as other external parties that influenced the process of implementing interventions for reducing hospital readmission.

**Patient needs and resources/intervention participant – patients’ confidence and compliance:** Detailed health needs assessment before discharge would facilitate case managers to plan suitable services for patients and caregivers.^24,29,30,32^ Eliciting input from patients and caregivers allowed service recipients to have a sense of ownership and collaboration,^29^ thus improving confidence and adherence to the interventions.^30,35^ On the other hand, poor communication between intervention providers, patients and caregivers would decrease patients’ confidence in the interventions,^26,30,32^ decreasing the compliance and fidelity of the interventions.

 However, some nurses suggested that patients’ self-reported information might not be accurate, and there is no reliable assessment tools for planning peri-discharge interventions.^25^ Also, some patients were unable to understand the discharge instructions or follow the plans completely.^24,29,30^ Nurses and case managers indicated that inadequate family, financial and social support would lower patients’ compliance with the prescribed intervention plan.^10,25,29,30,32^ Finally, excessive anxiety among some patients regarding discharge itself is another reason for rejecting the intervention.^24^ These observations suggests that not all patients are suitable to receive peri-discharge interventions.

**Cosmopolitanism:** In health systems which is not entirely tax funded, peri-discharge interventions may be resisted by external providers, such as community based primary care providers or social service institutions due to competition in the market.^10^ In fact, weak linkage between the hospital and external providers of primary care and social services is a key barrier in integrating different service components in peri-discharge interventions,^24,32^ as the nature peri-discharge intervention require inputs from different levels of health and social care at different settings.

**External policy and incentives:** External support to the hospital leading the implementation process, such as providing lists of primary and social care partners or patient education resources, can address implementation barriers which the hospital alone cannot tackle.^26,27,33^ Promulgation of financial penalties for hospitals with high readmission rates could urge leaders and providers to implement interventions for reducing readmission.^10,31^ The use of these policies requires a top-down change in regulation and reimbursement mechanisms, and how these would influence frontline providers’ action is unclear.^36^ It is suggested that the pros and cons of using these top-down mechanisms should be carefully considered, taking into account features of different health system contexts.^37^

## Discussion

 This systematic review of 13 qualitative studies identified barriers and facilitators to implementing peri-discharge interventions for reducing hospital readmission. Common barriers included (*i*) limited resources in terms of manpower and time, (*ii*) poor communication within the multidisciplinary implementation teams, (*iii*) incompatibility between additional requirements for intervention implementation and existing work demands, (*iv*) complicated implementation process in integrating service across organizations, (*v*) low practicality of supporting instruments, such as electronic health records, implementation checklists and service delivery guidelines, and (*vi*) implementation team members’ lack of understanding about interventions’ details and effectiveness. Common facilitators were (*i*) information sharing via regular meetings and timely communication within the multidisciplinary implementation teams, (*ii*) organizational culture that valued quality improvement and accountability, (*iii*) financial penalties for hospitals with high avoidable readmissions rates and external support offered via quality improvement programs and community resources, as well as (*iv*) senior leadership support.

###  Implications for Public and Policy

 Existing literature provided general insights on how barriers mentioned above may be addressed, specifically on the main issues which we have identified as overarching themes. The first theme is readiness and effort of the multidisciplinary team in implementing peri-discharge interventions. Aside from a common commitment from the team in changing current practice, establishment of internal support mechanisms within a multi-professional network is one of the keys for implementation success. A scoping review of 99 studies indicated that training, education, as well as audit and feedback among healthcare providers were the most common strategies for improving compliance towards new intervention implementation.^35^ Apart from providing clinical skills training, teamwork education would help the multidisciplinary team to value others’ perspectives, as well as to foster collegial trust and respect in the implementation process.^34^ Existing experience suggests that such educational program is effective in creating a positive culture of learning and collaboration within the implementation team.^34^ Healthcare providers’ social interaction skills can also be enhanced, which would then improve communications across team members in the complex process of implementation.^38^

 The second overarching theme is external stakeholders’ influence on peri-discharge interventions implementation. Echoing a previous systematic review of broader scope,^39^ we observed that a top-down imperative of financial penalties is effective in driving the implementation of readmission prevention interventions. While a retrospective cohort study showed that financial penalties for hospitals with high readmissions are associated with a significant reduction of 30-day and 1-year readmissions, such policy may also lead to negative unintended consequences.^40^ To avoid potential penalties, hospital management may “game the system” by increasing the percentage of patients placed on observation status instead of readmissions.^41^ They may even aggressively reduce necessary readmissions, which may result in increased mortality.^40^ With varying contexts and circumstances in different countries and healthcare systems, the application of financial penalties as an implementation intervention requires careful tailoring to avoid inadvertent harms to patients.

 The last overarching theme is rationality of the peri-discharge interventions, of which strategies to ensure operability, compatibility with the health system context and the usefulness of instruments and tools for facilitating the implementation processes are regarded as important determinants for success. Complexity and design quality of peri-discharge interventions are important barriers identified in this systematic review. One possible strategy to simplify complexity is to focus on core interventions components that are found to be critical in leading to better outcomes. Perceived complexity can also be reduced by designing a well-supported clinical pathway, or by breaking down the complex interventions into more manageable parts and adopting them incrementally.^42^ In addition, policymakers may place more emphasis on pilot testing the peri-discharge interventions using complexity reducing strategies described above, and subsequently fine-tune the interventions for better feasibility and adaptability prior to full-scale implementation.^43^ Finally, it is important to highlight that involvement of patients and caregivers should be regarded as a core part in the design of peri-discharge intervention, as mentioned in our overarching theme of external stakeholder consideration.

 A descriptive review of 70 cluster randomized controlled trials evaluating different implementation strategies for complex interventions indicated that the rationale and operational details of these strategies are often poorly reported.^44^ For example, details on who, where and when to provide different components of the implementation interventions were often omitted, limiting their real-world replicability. Efforts are needed to improve reporting in accordance with the Standards for Reporting Implementation Studies guideline.^45^

 In order to innovate implementation strategies that are tailored to local healthcare systems’ context, in the future researchers may consider mapping our CFIR based findings onto the Expert Recommendations for Implementation Change (ERIC).^46,47^ ERIC is an established catalogue of implementation strategies.^46^ With the use of CFIR-ERIC Implementation Strategy Matching Tool, some of the suggestions made in this systematic review may be augmented to innovate implementation strategies.^47^ For individual healthcare systems, implementation strategies may be adjusted and finalized using Delphi stakeholder consensus.^48^ Through this process, stakeholders-endorsed implementation strategies may be contextualized to meet local needs, thus facilitating the implementation of peri-discharge interventions in a relevant manner. This challenging implementation process could be led by implementation support practitioners, who should possess a wide range of skills including knowledge on service improvement practice, change process management, evidence-based practice facilitation, and issues regarding hospital readmission in the local healthcare context.^49^

###  Strengths and Limitations

 To ensure the methodological rigor of this systematic review, we applied an established methodological approach, including extensive literature search, methodological quality assessment and framework analysis. The use of CFIR also facilitated the categorization of barriers and facilitators that influenced the implementation.

 Ten out of thirteen studies included in this systematic review were conducted in the United States. Such lack of diversity limits the generalizability of our findings. Also, trustworthiness of our findings may be limited by methodological flaws among included studies. For example, six studies did not discuss why the certain qualitative research design was chosen, and more than half of the included studies performed poorly on considering and reporting relationship between researchers and participants. Bias could influence results if there were conflicts of interest between researchers and participants. In the future, qualitative research on this topic should report such relationships transparently, and justify research approach used. Indeed, the number of qualitative implementation research published is small relative to the large amount of literature describing peri-discharge interventions. More implementation research is needed especially outside of the US health system.

 For implementation problems, the Cochrane Collaboration currently recommends that qualitative and quantitative studies should be synthesized independently before integration.^50^ Only qualitative studies are synthesized in this systematic review, and future work should integrate our findings to published quantitative synthesis on peri-discharge intervention effectiveness.^51-53^ By using a logic model approach, such integration may inform the mechanisms of how implementation determinants may influence the delivery of different components of the complex peri-discharge interventions, which would eventually affect avoidable readmission incidence.^54^

## Conclusion

 This systematic review of qualitative findings synthesized barriers and facilitators to implementing peri-discharge interventions for reducing avoidable hospital readmission. Ensuring implementation fidelity, and active participation of patients and caregivers are key to reducing avoidable readmission successfully. This requires substantial commitment from both frontline providers and senior management given the complex nature of the intervention. We observed the importance of designing a well-supported pathway where responsibilities are clearly shared across partners. This demand managerial skills in promoting integrated care, as such interventions will always involve collaboration between hospital, primary care and social services. External resources support and financial mandates appeared to be key policy drivers for driving complex integrated care, as the former would ease additional burden of implementing new interventions, and the latter would influence sustainability of the healthcare organization. These implications are starting points for developing tailored implementation strategies for different healthcare systems via formative intervention mapping and consensus-seeking processes.

## Ethical issues

 All data for this study were obtained from existing publications, thus ethical approval was not required for this research.

## Competing interests

 Authors declare that they have no competing interests.

## Authors’ contributions

 VCC conceptualized the purpose of this systematic review. CCZ and CHW conducted literature search. BQF, CCZ, and FFH conducted literature selection. BQF, CCZ, and CHW carried out methodological quality assessment, data collection, data analysis, and synthesis. BQF and CCZ wrote the first draft of the manuscript, and revisions were made together with CHW, PN, CTH, EKY, and VCC. All authors approved the final version and revisions.

## Supplementary files


Supplementary file 1. Enhancing Transparency in Reporting the Synthesis of Qualitative Research: The ENTREQ Statement Checklist.

Supplementary file 2. Search Strategy and Results.

Supplementary file 3. Search Strategy and Results of Updated Literature Search.

Supplementary file 4. Flowchart of Updated Literature Search and Selection Process for Studies Published Between 2020 to Oct 2021.

Supplementary file 5. Methodological Quality of Thirteen Included Qualitative Studies.
