# Supplementary material for: Barriers and Facilitators to Implementing Interventions for Reducing Avoidable Hospital Readmission: Systematic Review of Qualitative Studies
Source: Int J Health Policy Manag. 2023 Feb 14;12:7089. doi: 10.34172/ijhpm.2023.7089 (PMC10125127; doi:10.34172/ijhpm.2023.7089)
Supplement: Supplementary file 2 — Search Strategy and Results. [file ijhpm-12-7089-s002.pdf]

Article title: Barriers and Facilitators to Implementing Interventions for Reducing Avoidable Hospital Readmission: Systematic Review of Qualitative Studies

Journal name: International Journal of Health Policy and Management (IJHPM)

Authors' information: Becky Q Fu[1], Claire CW Zhong[1], Charlene HL Wong[1], Fai Fai Ho[2], Per Nilsen[3], Chi Tim Hung[1], Eng Kiong Yeoh[1], Vincent CH Chung[1,2]*

[1]Centre for Health Systems and Policy Research, Jockey Club School of Public Health and Primary Care, The Chinese University of Hong Kong, Shatin, Hong Kong.

[2]School of Chinese Medicine, The Chinese University of Hong Kong, Shatin, Hong Kong.

[3]Department of Medicine, Health and Caring Sciences, Linköping University, Linköping, Sweden.

(*Corresponding author: vchung@cuhk.edu.hk)

Supplementary file 2. Search Strategy and Results

(i) MEDLINE from inception to March 24, 2020

#	Search Statement	Results
1	patient discharge/ or "hospital discharge".mp.	47203
2	1 and patient care planning/	756
3	(readmit* or readmission*).mp. or patient readmission/ or (re adj (admit* or admission* or hospital*)).mp. or rehospital*.mp. or postdischarge*.mp. or (post adj discharge).mp.	41163
4	3 and (intervention*.ti,ab. or interventional study.pt.)	7565
5	(discharge planning or "discharge instruction*" or "individuali?ed plan").mp.	3479
6	(aftercare or after care or ((discharge or care) adj2 bundle*)).mp.	12764
7	patient care management/ or primary health care/ or patient education as topic/ or "patient education".mp. or "care plan*1".mp. or "care pathway*".mp.	183016
8	continuity of patient care/ or geriatric assessment/ or mobility limitation/ or exp rehabilitation/ or exp physical therapy modalities/	345244

9	(telephone or telemonitor* or telemedicine).mp. or home care services/ or house calls/ or support*.mp. or physical fitness.mp. or exercise/ or exercise therapy/ or activities of daily living/ or self care/	9568855
10	((care or case or self) adj manage*).mp. or disability evaluation/ or "social work*".mp. or primary care team/ or community health services/ or "care transition".mp. or counseling.mp. or coaching.mp. or remind*.mp. or health services for the elderly/	265159
11	"Appointments and Schedules"/	8835
12	risk reduction behavior/ or risk assessment/ or risk factors/ or health knowledge attitudes/ or patient satisfaction/ or "home visit*".mp. or home nursing.mp. or pharmacist*.mp. or medication reconciliation/ or (hospital adj2 home).mp. or barrier*.mp. or family practice/ or physicians, family/ or "follow-up".mp.	2478760
13	(socioeconomic factors or literacy).mp. or marital status/ or "social adj support".mp.	181118
14	2 or 4 or 5 or 6 or 7 or 8 or 9 or 10 or 11 or 12 or 13	11460326
15	(30 day or thirty day).mp.	36207
16	3 and 14 and 15	4316
17	interview:.tw. or px.fs. or exp health services administration/	4016146
18	16 and 17	3293

(ii) EMBASE from inception to March 24, 2020

#	Search Statement	Results
1	patient discharge/ or "hospital discharge".mp.	133402
2	1 and patient care planning/	701
3	(readmit* or readmission*).mp. or patient readmission/ or (re adj (admit* or admission* or hospital*)).mp. or rehospital*.mp. or postdischarge*.mp. or (post adj discharge).mp.	95881
4	3 and (intervention*.ti,ab. or interventional study.pt.)	17982
5	(discharge planning or "discharge instruction*" or "individuali?ed plan").mp.	5951
6	(aftercare or after care or ((discharge or care) adj2 bundle*)).mp.	12142

7	patient care management/ or primary health care/ or patient education as topic/ or "patient education".mp. or "care plan*1".mp. or "care pathway*".mp.	457180
8	continuity of patient care/ or geriatric assessment/ or mobility limitation/ or exp rehabilitation/ or exp physical therapy modalities/	715068
9	(telephone or telemonitor* or telemedicine).mp. or home care services/ or house calls/ or support*.mp. or physical fitness.mp. or exercise/ or exercise therapy/ or activities of daily living/ or self care/	2489872
10	((care or case or self) adj manage*).mp. or disability evaluation/ or "social work*".mp. or primary care team/ or community health services/ or "care transition".mp. or counseling.mp. or coaching.mp. or remind*.mp. or health services for the elderly/	479238
11	"Appointments and Schedules"/	40427
12	risk reduction behavior/ or risk assessment/ or risk factors/ or health knowledge attitudes/ or patient satisfaction/ or "home visit*".mp. or home nursing.mp. or pharmacist*.mp. or medication reconciliation/ or (hospital adj2 home).mp. or barrier*.mp. or family practice/ or physicians, family/ or "follow-up".mp.	3691299
13	(socioeconomic factors or literacy).mp. or marital status/ or "social adj support".mp.	87321
14	2 or 4 or 5 or 6 or 7 or 8 or 9 or 10 or 11 or 12 or 13	6595088
15	(30 day or thirty day).mp.	73257
16	3 and 14 and 15	8880
17	(interview: or qualitative).tw. or exp health care organization/	2044106
18	16 and 17	2439

(iii) PsycInfo from inception to March 24, 2020

#	Search Statement	Results
1	patient discharge/ or "hospital discharge".mp.	5464
2	1 and patient care planning/	21
3	(readmit* or readmission*).mp. or patient readmission/ or (re adj (admit* or admission* or hospital*)).mp. or rehospital*.mp. or postdischarge*.mp. or (post adj discharge).mp.	7249
4	3 and (intervention*.ti,ab. or interventional study.pt.)	1579
5	(discharge planning or "discharge instruction*" or "individuali?ed plan").mp.	1253
6	(aftercare or after care or ((discharge or care) adj2 bundle*)).mp.	3989

7	patient care management/ or primary health care/ or patient education as topic/ or "patient education".mp. or "care plan*1".mp. or "care pathway*".mp.	24063
8	continuity of patient care/ or geriatric assessment/ or mobility limitation/ or exp rehabilitation/ or exp physical therapy modalities/	48258
9	(telephone or telemonitor* or telemedicine).mp. or home care services/ or house calls/ or support*.mp. or physical fitness.mp. or exercise/ or exercise therapy/ or activities of daily living/ or self care/	714400
10	((care or case or self) adj manage*).mp. or disability evaluation/ or "social work*".mp. or primary care team/ or community health services/ or "care transition".mp. or counseling.mp. or coaching.mp. or remind*.mp. or health services for the elderly/	188018
11	"Appointments and Schedules"/	0
12	risk reduction behavior/ or risk assessment/ or risk factors/ or health knowledge attitudes/ or patient satisfaction/ or "home visit*".mp. or home nursing.mp. or pharmacist*.mp. or medication reconciliation/ or (hospital adj2 home).mp. or barrier*.mp. or family practice/ or physicians, family/ or "follow-up".mp.	281676
13	(socioeconomic factors or literacy).mp. or marital status/ or "social adj support".mp.	71320
14	2 or 4 or 5 or 6 or 7 or 8 or 9 or 10 or 11 or 12 or 13	1154919
15	(30 day or thirty day).mp.	3124
16	3 and 14 and 15	265
17	experience:.mp. or interview:.tw. or qualitative:.tw.	943283
18	16 and 17	49

(iv) Global Health from inception to March 24, 2020

#	Search Statement	Results
1	patient discharge/ or "hospital discharge".mp.	4073
2	1 and patient care planning/	0
3	(readmit* or readmission*).mp. or patient readmission/ or (re adj (admit* or admission* or hospital*)).mp. or rehospital*.mp. or postdischarge*.mp. or (post adj discharge).mp.	4226
4	3 and (intervention*.ti,ab. or interventional study.pt.)	759
5	(discharge planning or "discharge instruction*" or "individuali?ed plan").mp.	178
6	(aftercare or after care or ((discharge or care) adj2 bundle*)).mp.	458

7	patient care management/ or primary health care/ or patient education as topic/ or "patient education".mp. or "care plan*1".mp. or "care pathway*".mp.	15988
8	continuity of patient care/ or geriatric assessment/ or mobility limitation/ or exp rehabilitation/ or exp physical therapy modalities/	1285
9	(telephone or telemonitor* or telemedicine).mp. or home care services/ or house calls/ or support*.mp. or physical fitness.mp. or exercise/ or exercise therapy/ or activities of daily living/ or self care/	239495
10	((care or case or self) adj manage*).mp. or disability evaluation/ or "social work*".mp. or primary care team/ or community health services/ or "care transition".mp. or counseling.mp. or coaching.mp. or remind*.mp. or health services for the elderly/	29879
11	"Appointments and Schedules"/	0
12	risk reduction behavior/ or risk assessment/ or risk factors/ or health knowledge attitudes/ or patient satisfaction/ or "home visit*".mp. or home nursing.mp. or pharmacist*.mp. or medication reconciliation/ or (hospital adj2 home).mp. or barrier*.mp. or family practice/ or physicians, family/ or "follow-up".mp.	385446
13	(socioeconomic factors or literacy).mp. or marital status/ or "social adj support".mp.	10683
14	2 or 4 or 5 or 6 or 7 or 8 or 9 or 10 or 11 or 12 or 13	612823
15	(30 day or thirty day).mp.	5512
16	3 and 14 and 15	292
17	(experience: or interview: or qualitative:).mp. [mp=abstract, title, original title, broad terms, heading words, identifiers, cabicodes]	223050
18	16 and 17	34
