# Supplementary material for: Barriers and Facilitators to Implementing Interventions for Reducing Avoidable Hospital Readmission: Systematic Review of Qualitative Studies
Source: Int J Health Policy Manag. 2023 Feb 14;12:7089. doi: 10.34172/ijhpm.2023.7089 (PMC10125127; doi:10.34172/ijhpm.2023.7089)
Supplement: Supplementary file 3 — Search Strategy and Results of Updated Literature Search. [file ijhpm-12-7089-s003.pdf]

Article title: Barriers and Facilitators to Implementing Interventions for Reducing Avoidable Hospital Readmission: Systematic Review of Qualitative Studies

Journal name: International Journal of Health Policy and Management (IJHPM)

Authors' information: Becky Q Fu[1], Claire CW Zhong[1], Charlene HL Wong[1], Fai Fai Ho[2], Per Nilsen[3], Chi Tim Hung[1], Eng Kiong Yeoh[1], Vincent CH Chung[1,2]*

[1]Centre for Health Systems and Policy Research, Jockey Club School of Public Health and Primary Care, The Chinese University of Hong Kong, Shatin, Hong Kong.

[2]School of Chinese Medicine, The Chinese University of Hong Kong, Shatin, Hong Kong.

[3]Department of Medicine, Health and Caring Sciences, Linköping University, Linköping, Sweden.

(*Corresponding author: vchung@cuhk.edu.hk)

Supplementary file 3. Search Strategy and Results of Updated Literature Search

MEDLINE from 2020 to October 4, 2021

#	Search Statement	Results
1	patient discharge/ or "hospital discharge".mp.	54816
2	1 and patient care planning/	774
3	(readmit* or readmission*).mp. or patient readmission/ or (re adj (admit* or admission* or hospital*)).mp. or rehospital*.mp. or postdischarge*.mp. or (post adj discharge).mp.	50412
4	3 and (intervention*.ti,ab. or interventional study.pt.)	9672
5	(discharge planning or "discharge instruction*" or "individuali?ed plan").mp.	3912
6	(aftercare or after care or ((discharge or care) adj2 bundle*)).mp.	15209
7	patient care management/ or primary health care/ or patient education as topic/ or "patient education".mp. or "care plan*1".mp. or "care pathway*".mp.	198414
8	continuity of patient care/ or geriatric assessment/ or mobility limitation/ or exp rehabilitation/ or exp physical therapy modalities/	377365

9	(telephone or telemonitor* or telemedicine).mp. or home care services/ or house calls/ or support*.mp. or physical fitness.mp. or exercise/ or exercise therapy/ or activities of daily living/ or self care/	10330119
10	((care or case or self) adj manage*).mp. or disability evaluation/ or "social work*".mp. or primary care team/ or community health services/ or "care transition".mp. or counseling.mp. or coaching.mp. or remind*.mp. or health services for the elderly/	289701
11	"Appointments and Schedules"/	9435
12	risk reduction behavior/ or risk assessment/ or risk factors/ or health knowledge attitudes/ or patient satisfaction/ or "home visit*".mp. or home nursing.mp. or pharmacist*.mp. or medication reconciliation/ or (hospital adj2 home).mp. or barrier*.mp. or family practice/ or physicians, family/ or "follow-up".mp.	2729582
13	(socioeconomic factors or literacy).mp. or marital status/ or "social adj support".mp.	198412
14	2 or 4 or 5 or 6 or 7 or 8 or 9 or 10 or 11 or 12 or 13	12389620
15	(30 day or thirty day).mp.	44554
16	3 and 14 and 15	6046
17	interview:.tw. or px.fs. or exp health services administration/	4407772
18	16 and 17	4486
19	limit 18 to yr="2020 -Current"	917

(i) EMBASE from 2020 to October 4, 2021

#	**Search Statement**	**Results**
1	patient discharge/ or "hospital discharge".mp.	158309
2	1 and patient care planning/	723
3	(readmit* or readmission*).mp. or patient readmission/ or (re adj (admit* or admission* or hospital*)).mp. or rehospital*.mp. or postdischarge*.mp. or (post adj discharge).mp.	116274
4	3 and (intervention*.ti,ab. or interventional study.pt.)	22288
5	(discharge planning or "discharge instruction*" or "individuali?ed plan").mp.	6681
6	(aftercare or after care or ((discharge or care) adj2 bundle*)).mp.	13731

7	patient care management/ or primary health care/ or patient education as topic/ or "patient education".mp. or "care plan*1".mp. or "care pathway*".mp.	501606
8	continuity of patient care/ or geriatric assessment/ or mobility limitation/ or exp rehabilitation/ or exp physical therapy modalities/	800705
9	(telephone or telemonitor* or telemedicine).mp. or home care services/ or house calls/ or support*.mp. or physical fitness.mp. or exercise/ or exercise therapy/ or activities of daily living/ or self care/	2812494
10	((care or case or self) adj manage*).mp. or disability evaluation/ or "social work*".mp. or primary care team/ or community health services/ or "care transition".mp. or counseling.mp. or coaching.mp. or remind*.mp. or health services for the elderly/	531268
11	"Appointments and Schedules"/	41788
12	risk reduction behavior/ or risk assessment/ or risk factors/ or health knowledge attitudes/ or patient satisfaction/ or "home visit*".mp. or home nursing.mp. or pharmacist*.mp. or medication reconciliation/ or (hospital adj2 home).mp. or barrier*.mp. or family practice/ or physicians, family/ or "follow-up".mp.	4214677
13	(socioeconomic factors or literacy).mp. or marital status/ or "social adj support".mp.	102484
14	2 or 4 or 5 or 6 or 7 or 8 or 9 or 10 or 11 or 12 or 13	7440012
15	(30 day or thirty day).mp.	87274
16	3 and 14 and 15	11607
17	(interview: or qualitative).tw. or exp health care organization/	2251687
18	16 and 17	3024
19	limit 18 to yr="2020 -Current"	643

(ii) PsycInfo from 2020 to October 4, 2021

#	**Search Statement**	**Results**
1	patient discharge/ or "hospital discharge".mp.	6078
2	1 and patient care planning/	28
3	(readmit* or readmission*).mp. or patient readmission/ or (re adj (admit* or admission* or hospital*)).mp. or rehospital*.mp. or postdischarge*.mp. or (post adj discharge).mp.	8017

4	3 and (intervention*.ti,ab. or interventional study.pt.)	1833
5	(discharge planning or "discharge instruction*" or "individuali?ed plan").mp.	1405
6	(aftercare or after care or ((discharge or care) adj2 bundle*)).mp.	4190
7	patient care management/ or primary health care/ or patient education as topic/ or "patient education".mp. or "care plan*1".mp. or "care pathway*".mp.	26319
8	continuity of patient care/ or geriatric assessment/ or mobility limitation/ or exp rehabilitation/ or exp physical therapy modalities/	52367
9	(telephone or telemonitor* or telemedicine).mp. or home care services/ or house calls/ or support*.mp. or physical fitness.mp. or exercise/ or exercise therapy/ or activities of daily living/ or self care/	783858
10	((care or case or self) adj manage*).mp. or disability evaluation/ or "social work*".mp. or primary care team/ or community health services/ or "care transition".mp. or counseling.mp. or coaching.mp. or remind*.mp. or health services for the elderly/	200704
11	"Appointments and Schedules"/	0
12	risk reduction behavior/ or risk assessment/ or risk factors/ or health knowledge attitudes/ or patient satisfaction/ or "home visit*".mp. or home nursing.mp. or pharmacist*.mp. or medication reconciliation/ or (hospital adj2 home).mp. or barrier*.mp. or family practice/ or physicians, family/ or "follow-up".mp.	313002
13	(socioeconomic factors or literacy).mp. or marital status/ or "social adj support".mp.	78070
14	2 or 4 or 5 or 6 or 7 or 8 or 9 or 10 or 11 or 12 or 13	1260492
15	(30 day or thirty day).mp.	3672
16	3 and 14 and 15	346
17	experience:.mp. or interview:.tw. or qualitative:.tw.	1034102
18	16 and 17	71
19	limit 18 to yr="2020 -Current"	20

(iii) Global Health from 2020 to October 4, 2021

#	Search Statement	Results
1	patient discharge/ or "hospital discharge".mp.	4797
2	1 and patient care planning/	0

3	(readmit* or readmission*).mp. or patient readmission/ or (re adj (admit* or admission* or hospital*)).mp. or rehospital*.mp. or postdischarge*.mp. or (post adj discharge).mp.	5338
4	3 and (intervention*.ti,ab. or interventional study.pt.)	1007
5	(discharge planning or "discharge instruction*" or "individuali?ed plan").mp.	213
6	(aftercare or after care or ((discharge or care) adj2 bundle*)).mp.	568
7	patient care management/ or primary health care/ or patient education as topic/ or "patient education".mp. or "care plan*1".mp. or "care pathway*".mp.	19992
8	continuity of patient care/ or geriatric assessment/ or mobility limitation/ or exp rehabilitation/ or exp physical therapy modalities/	1839
9	(telephone or telemonitor* or telemedicine).mp. or home care services/ or house calls/ or support*.mp. or physical fitness.mp. or exercise/ or exercise therapy/ or activities of daily living/ or self care/	281858
10	((care or case or self) adj manage*).mp. or disability evaluation/ or "social work*".mp. or primary care team/ or community health services/ or "care transition".mp. or counseling.mp. or coaching.mp. or remind*.mp. or health services for the elderly/	35066
11	"Appointments and Schedules"/	0
12	risk reduction behavior/ or risk assessment/ or risk factors/ or health knowledge attitudes/ or patient satisfaction/ or "home visit*".mp. or home nursing.mp. or pharmacist*.mp. or medication reconciliation/ or (hospital adj2 home).mp. or barrier*.mp. or family practice/ or physicians, family/ or "follow-up".mp.	456405
13	(socioeconomic factors or literacy).mp. or marital status/ or "social adj support".mp.	13907
14	2 or 4 or 5 or 6 or 7 or 8 or 9 or 10 or 11 or 12 or 13	722473
15	(30 day or thirty day).mp.	7039
16	3 and 14 and 15	422
17	(experience: or interview: or qualitative:).mp. [mp=abstract, title, original title, broad terms, heading words, identifiers, cabicodes]	262630
18	16 and 17	58
19	limit 18 to yr="2020 -Current"	21
