# Supplementary material for: Barriers and Facilitators to Implementing Interventions for Reducing Avoidable Hospital Readmission: Systematic Review of Qualitative Studies
Source: Int J Health Policy Manag. 2023 Feb 14;12:7089. doi: 10.34172/ijhpm.2023.7089 (PMC10125127; doi:10.34172/ijhpm.2023.7089)
Supplement: Supplementary file 4 — Flowchart of Updated Literature Search and Selection Process for Studies Published Between 2020 to Oct 2021. [file ijhpm-12-7089-s004.pdf]

Article title: Barriers and Facilitators to Implementing Interventions for Reducing Avoidable Hospital Readmission: Systematic Review of Qualitative Studies

Journal name: International Journal of Health Policy and Management (IJHPM)

Authors' information: Becky Q Fu[1], Claire CW Zhong[1], Charlene HL Wong[1], Fai Fai Ho[2], Per Nilsen[3], Chi Tim Hung[1], Eng Kiong Yeoh[1], Vincent CH Chung[1,2]*

[1]Centre for Health Systems and Policy Research, Jockey Club School of Public Health and Primary Care, The Chinese University of Hong Kong, Shatin, Hong Kong.

[2]School of Chinese Medicine, The Chinese University of Hong Kong, Shatin, Hong Kong.

[3]Department of Medicine, Health and Caring Sciences, Linköping University, Linköping, Sweden.

(*Corresponding author: vchung@cuhk.edu.hk)

Supplementary file 4. Flowchart of Updated Literature Search and Selection Process for Studies Published Between 2020 to Oct 2021

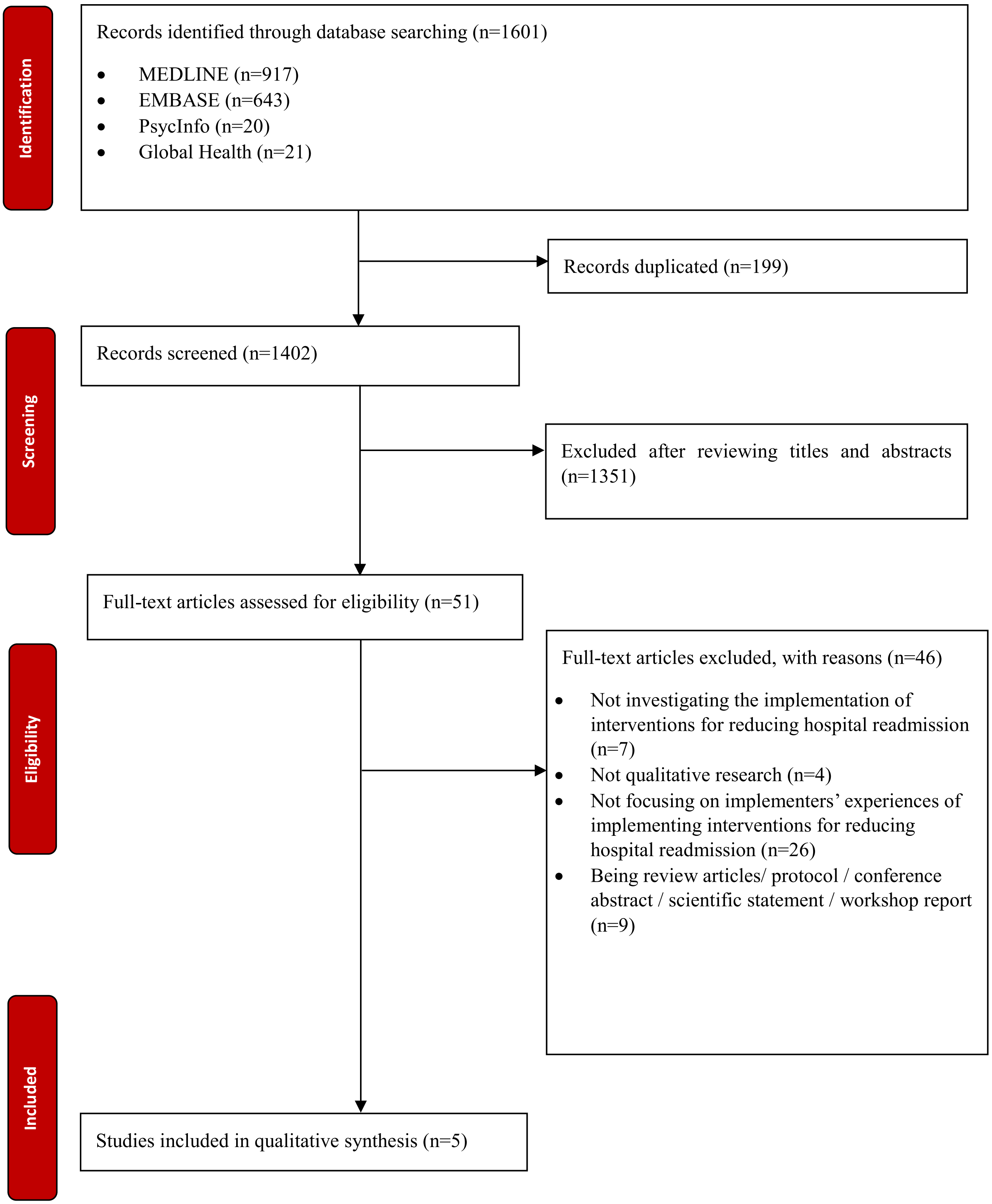
Identification
Records identified through database searching (n=1601)
MEDLINE (n=917)
EMBASE (n=643)
PsycInfo (n=20)
Global Health (n=21)
Records duplicated (n=199)
Screening
Records screened (n=1402)
Excluded after reviewing titles and abstracts (n=1351)
Eligibility
Full-text articles assessed for eligibility (n=51)
Full-text articles excluded, with reasons (n=46)
Not investigating the implementation of interventions for reducing hospital readmission (n=7)
Not qualitative research (n=4)
Not focusing on implementers' experiences of implementing interventions for reducing hospital readmission (n=26)
Being review articles/ protocol / conference abstract / scientific statement / workshop report (n=9)
Included
Studies included in qualitative synthesis (n=5)
