# Supplementary material for: Barriers and Facilitators to Implementing Interventions for Reducing Avoidable Hospital Readmission: Systematic Review of Qualitative Studies
Source: Int J Health Policy Manag. 2023 Feb 14;12:7089. doi: 10.34172/ijhpm.2023.7089 (PMC10125127; doi:10.34172/ijhpm.2023.7089)
Supplement: Supplementary file 5 — Methodological Quality of Thirteen Included Qualitative Studies. [file ijhpm-12-7089-s005.pdf]

Article title: Barriers and Facilitators to Implementing Interventions for Reducing Avoidable Hospital Readmission: Systematic Review of Qualitative Studies

Journal name: International Journal of Health Policy and Management (IJHPM)

Authors' information: Becky Q Fu[1], Claire CW Zhong[1], Charlene HL Wong[1], Fai Fai Ho[2], Per Nilsen[3], Chi Tim Hung[1], Eng Kiong Yeoh[1], Vincent CH Chung[1,2]*

[1]Centre for Health Systems and Policy Research, Jockey Club School of Public Health and Primary Care, The Chinese University of Hong Kong, Shatin, Hong Kong.

[2]School of Chinese Medicine, The Chinese University of Hong Kong, Shatin, Hong Kong.

[3]Department of Medicine, Health and Caring Sciences, Linköping University, Linköping, Sweden.

(*Corresponding author: vchung@cuhk.edu.hk)

Supplementary file 5. Methodological Quality of Thirteen Included Qualitative Studies

First author and publication year	CASP qualitative research checklist items									
	1	2	3	4	5	6	7	8	9	10
**Danielsen[23] 2020**	Y	Y	Y	N/A	Y	N	Y	Y	Y	Y

First author and publication year	CASP qualitative research checklist items									
	**1**	**2**	**3**	**4**	**5**	**6**	**7**	**8**	**9**	**10**
**Lai[24] 2021**	Y	Y	N/A	Y	Y	N	Y	Y	Y	Y
**Lee[25] 2013**	Y	Y	Y	Y	Y	Y	Y	Y	Y	Y
**Lehn[9] 2018**	Y	Y	Y	N/A	Y	N	Y	Y	Y	Y
**Machta[26] 2016**	Y	Y	Y	Y	Y	Y	Y	Y	Y	Y
**Meehan[27] 2017**	Y	Y	N/A	Y	Y	N	N	Y	Y	Y
**Meehan[28] 2015**	Y	Y	N/A	Y	Y	N	N	N/A	Y	Y
**Misra-Hebert[29] 2021**	Y	Y	Y	Y	Y	N	N	Y	Y	Y
**Mitchell[10] 2016**	Y	Y	Y	N/A	Y	N	N	Y	Y	Y
**Nation[30] 2019**	Y	Y	Y	Y	Y	Y	Y	Y	Y	Y
**Rask[31] 2017**	Y	Y	N/A	N/A	Y	Y	N	Y	Y	Y
**Riddle[32] 2020**	Y	Y	N/A	Y	Y	N	Y	Y	Y	Y
**Romaire[33] 2020**	Y	Y	N/A	Y	Y	N	Y	Y	Y	Y
**# of Yes (%)**	13 (100)	13 (100)	7 (54)	9 (69)	13 (100)	4 (31)	8 (62)	12 (92)	13 (100)	13 (100)

Keys: N: no; N/A: Can’t tell; Y: yes (studies fulfilling the criteria); # of Yes: number of yes; CASP: Critical Appraisal Skills Programme;

CASP qualitative research checklist item: 1. Was there a clear statement of the aims of the research? 2. Is a qualitative methodology appropriate? 3. Was the research design appropriate to address the aims of the research? 4. Was the recruitment strategy appropriate to the aims of the research? 5. Was the data collected in a way that addressed the research issue? 6. Has the relationship between researcher and participants been adequately considered? 7. Have ethical issues been taken into consideration? 8. Was the data analysis sufficiently rigorous? 9. Is there a clear statement of findings? 10. How valuable is the research?
